# Relationship of Choroidal Vasculature and Choriocapillaris Flow With Alterations of Salivary α-Amylase Patterns in Central Serous Chorioretinopathy

**DOI:** 10.1167/iovs.62.15.19

**Published:** 2021-12-21

**Authors:** Fabio Scarinci, Francesca Romana Patacchioli, Eliana Costanzo, Mariacristina Parravano

**Affiliations:** 1 IRCCS – Fondazione Bietti, Rome, Italy

**Keywords:** central serous chorioretinopathy, salivary α-amylase diurnal pattern, choroidal vascularity index, flow signal void area, optical coherence tomography angiography

## Abstract

**Purpose:**

An abnormality in choroidal vasculature is a known factor in the pathogenesis of central serous chorioretinopathy (CSC), a chorioretinal disease affecting mostly middle-aged males. The purpose of the present study was to investigate the role of the autonomic nervous system (ANS) in the pathophysiology of CSC.

**Methods:**

This was a cross-sectional observational study in which characteristic choroidal vasculature metrics were assessed by measuring the subfoveal choroidal thickness (FCT) and the choroidal vascularity index (CVI) using the imaging technique of enhanced-depth imaging spectral-domain optical coherence tomography (EDI-SD-OCT). Furthermore, flow signal void area features were also evaluated in the study population using OCT angiography (OCTA). Diurnal patterns of salivary α-amylase (a-AMY) production, proposed as a marker of autonomic activity, were assessed in an adult male study population affected by acute naïve CSC in comparison with matched healthy controls.

**Results:**

Results include an overall higher diurnal output of salivary a-AMY production, which is in line with the phenomenon of a sympathetic “drive” playing a role in the pathophysiology of CSC, and a flattened diurnal percentage variation in α-AMY in CSC-affected subjects. Furthermore, Pearson's coefficient test revealed statistically significant correlations between salivary α-AMY diurnal percentage variation and selected choroidal imaging biomarkers (FCT, CVI, and flow signal void area). Finally, multiple linear regression analysis identified salivary α-AMY diurnal percentage production as the sole predictor of the CVI and flow signal void area in the study population.

**Conclusions:**

Autonomic nervous system dysregulation was highlighted in CSC patients.

An abnormality in choroidal circulation with involvement of the retinal pigment epithelium (RPE) is widely accepted as one of the main pathophysiological mechanisms playing a role in the development of central serous chorioretinopathy (CSC).^1^ The introduction of new technologies such as optical coherence tomography (OCT) with the enhanced-depth imaging (EDI) technique, swept-source (SS) OCT, and OCT angiography (OCTA) have substantially improved our capability in qualitatively and quantitatively evaluating the assessment of choroidal microvasculature, providing a deeper analysis of the complex choroidal vascular system in different pathophysiological conditions.^2^^,^^3^ Therefore, in the present study, choroidal vasculature metrics were assessed by measuring the subfoveal choroidal thickness (FCT) and the choroidal vascularity index (CVI), a tool with binarization of EDI-SD-OCT images used to quantitatively measure and analyze the choroidal vascular system in both healthy and CSC-affected eyes.^4^ Furthermore, to gather information on the choriocapillaris status, flow signal void features were also evaluated in the study population using OCTA.^5^

Choroidal vessels are under the control of the autonomic nervous system (ANS), and evidence in the literature recognizes that autonomic dysfunction in CSC, causing the inability of choroidal vessels to maintain homeostasis and leading to choroidal hyperperfusion, would ultimately result in subretinal fluid accumulation.^6^^,^^7^ There is an established belief that CSC is related to type A personality, anxiety and even stress system dysregulation.^8^^–^^14^ Several methods evaluating ANS tone and reactivity have been used, including measures of plasma levels of norepinephrine.^15^

Summarizing the existing literature, early studies have proposed salivary α-amylase (α-AMY) assessments as a proxy measure of norepinephrine release that may subsequently elicit the release of α-AMY by the salivary glands, given that branches of the sympathetic and parasympathetic nerves are distributed in those.^16^^–^^21^ The ANS is one of the main adaptive systems in humans and shows a distinct circadian rhythm with sympathetic activity progressively increasing during the day.^22^ Similarly, salivary α-AMY secretion has a diurnal pattern that decreases immediately after awakening and is followed by a steady rise toward the afternoon and the evening.^23^^–^^25^

It has been suggested that the ANS shows hyperactivity in CSC patients, leading to higher salivary α-AMY levels in comparison with a control group.^14^ Therefore, in adult male subjects newly diagnosed with an acute episode of idiopathic CSC and matched healthy controls, two distinct measures of the diurnal salivary α-AMY secretion patterns were computed:

a) the area under the curve with respect to the ground (AUC_G_) of diurnal production, calculated using the salivary samples collected at 08:00 h, 12:00 h, and 20:00 h,^26^ and
b) the evaluation of the morning/evening diurnal percentage variation of salivary α-AMY.^25^^,^^27^

Purpose of the present study was to explore interdependencies of these salivary markers of ANS functioning (salivary α-AMY AUC_G_ and diurnal percentage variation) with all three selected biomarkers of CSC imaging (FCT, CVI, and flow signal void area) assessed in the adult male study population affected by acute naïve CSC in comparison with matched healthy controls. Therefore Pearson's correlation test was used to show any correlation between variables, and multiple linear regression was finally used to predict dependent variables.

## Materials and Methods

### Study Population

The Central Ethical Committee formally approved this cross-sectional observational study for Lazio, Italy (protocol no. 4327/April 18, 2018). In the a priori sample size calculation, we previously estimated that at least 28 subjects (14 per group) were required to detect a mean absolute difference of approximately 25% for the expected changes in diurnal salivary α-AMY production between the control and CSC groups, with α = 0.01, β = 0.2 and statistical power of 80%.^28^

Eighteen Caucasian male subjects aged 40 to 60 years who consecutively attended the outpatient clinic of the Retina Medical Service at Bietti Foundation from September 1, 2018, to December 15, 2019, were included in the study. They were newly diagnosed with an acute episode of idiopathic CSC, characterized by localized neurosensory retinal detachment with leakage at the level of the retinal RPE and with subretinal fluid, respectively, confirmed by fluorescein angiography and spectral-domain optical coherence tomography (SD-OCT) B-scan (Heidelberg Engineering, Heidelberg, Germany).^29^^–^^31^ They had unilateral clinical disease.

Full ophthalmological examination was also carried out for each of the participants of the study. Specifically, best-corrected visual acuity (BCVA) was evaluated using Early Treatment of Diabetic Retinopathy Study (ETDRS) letters. The presence of subretinal fluid was defined on the ETDRS grid; the central subfield was considered 1 mm in diameter centered around the center point of the fovea, whereas the inner ring circle has a diameter of 3 mm. Indocyanine green angiography was performed to explore choroidal vascular hyperpermeability and exclude occult choroidal neovascularization.^32^

Eighteen age-matched controls were recruited among Bietti Foundation employers/subjects accompanying patients to medical services at Bietti Foundation who did not present any ocular or retinal abnormalities, as shown by the ophthalmological examination and SD-OCT evaluation. Exclusion criteria were chronic CSC (duration of visual symptoms more than 12 weeks) or recurrent CSC, and the presence of choroidal neovascularization, uveitis history, optic disc edema, choroidal infiltrates, cotton wool spots, or retinal hemorrhages were excluded.

Furthermore, subjects and patients with high myopia or hyperopia (greater than −6 or +3 diopters [D] of refractive error) were also excluded. Metabolic, cardiovascular, and endocrine diseases, as well as smoking habits, were considered as exclusion criteria.

None of the study participants had received any steroidal anti-inflammatory or immunosuppressive drug in the previous 12 months, nor had they received any vasoactive substances drugs that could have influenced α-AMY production (e.g., antihypertensive, antipsychotic and thyroid agents). Furthermore, patients with a history of alcohol abuse and alcohol dependence, as well as having high caffeine intakes, were excluded as well?.

### SD-OCT Scan Protocol

The SD OCT images were obtained by using Spectralis OCT (Heidelberg Engineering, Heidelberg Germany). The scan protocol consisted of OCT raster scans obtained in the macular region, with a volume scan of 20°× 20° containing 25 B-scans centered on the fovea. The EDI-OCT set was used to better visualize the choroid-sclera interface.

Using an EDI technique, the measurement of the subfoveal choroidal thickness (FCT) was assessed at the B-scan passing through the foveal center, defined as the vertical distance between the outer border of the RPE and the choroid-sclera interface. Scans with signal strength of ≥6 were used for analysis.

To evaluate interrater and intrarater observer variability for FCT measurement, the same set of images was analyzed by two expert graders (ophthalmologists: E.C. and F.S.), and the intraclass correlation coefficients (ICCs) were calculated for statistical analyses: ICC <3.5% indicates poor agreement.^33^ The ICC for the present study was 0.87 for intrarater agreement and 0.89 for inter-rater agreement.

For the choroidal evaluation, the CVI, a quantitative OCT parameter, was calculated as follows:^4^ a raster EDI-OCT scan passing through the fovea was exported and analyzed by using ImageJ software version 1.50 (National Institutes of Health, Bethesda, MD, USA), as previously described.^34^^,^^35^ The total subfoveal scan was selected using a polygon tool and added to the region of interest manager to identify the total choroidal area. The image was converted into an 8-bit image, binarized using the Niblack automatic local threshold, converted into red, green, blue color images; dark pixels, measured to calculate the luminal choroidal area value, were selected using the color threshold tool and then added to the region of interest manager; white pixels corresponded to the stromal choroidal area; CVI was finally calculated as the ratio between luminal choroidal area and total choroidal area .^35^

### SS-OCTA Scan Protocol

Patients underwent SS-OCTA imaging using the PLEX Elite 9000 device using a swept laser source with a central wavelength of 1050 nm and a bandwidth of 100.^36^ This instrument has an axial resolution of approximately 5 microns and a lateral resolution estimated at approximately 14 µm. For the study eye, OCTA images using the 3 × 3 mm scan pattern centered on the fovea were acquired. Also, scan images with a signal strength index of ≥6 (a measurement in a scale of 0 to 10 indicating the level of retinal tissue signal with respect to the noise or background level in OCT data), as recommended by manufacturer. Images including detachments of epithelium pigmented (DEP) were excluded from the analysis.

For the evaluation of the choriocapillaris (CC), the total flow signal void area was measured. The total flow signal void area represents the total area of CC vascular dropouts (absence of flow or flow less than the slowest detectable threshold) as a percentage of each analyzed area.^37^^,^^38^ For each included eye, images of the superficial capillary plexus (SCP) and CC face OCTA, automatically segmented by the PlexElite 9000 (Zeiss, Oberkochen, Germany), were exported and analyzed using ImageJ software version 1.50 (National Institutes of Health). The automatic SCP slab was segmented between the internal limiting membrane and inner plexiform layer.^3^ The 15 mm–thick CC slab started 16 mm below the RPE/Bruch's membrane complex.^39^ All scans were checked for segmentation errors before image processing. The enface SCP image was opened in ImageJ, and the “Max Entropy” threshold was applied to visualize only the greater superficial retinal vessels (causing shadowing and artifacts).^2^

The CC images were binarized with the Phansalkar method and processed with the analyze particles tool to obtain the total flow signal void area.^39^ The two obtained images (CC enface image and image identifying superficial vessels) were merged to eliminate potentially confounding shadow or projection artifacts, as previously shown.^2^^,^^37^^,^^38^ The images were processed with the “Analyze Particles” command to measure the total flow signal voids.

### Experimental Procedure

Written informed consent was obtained from all participants during a preliminary informative meeting. During a subsequent experimental session, upon arrival (between 09:00 h and 12:00 h), somatic and clinical characteristics of the study population were collected, and subjects were taught how to collect their saliva at home (they were asked to avoid food, coffee, tooth brushing, and any physical exercise for at least 30 minutes before each saliva collection.^40^^,^^41^

Home diurnal saliva collection was scheduled at several timepoints on a single sampling day: 60 minutes after awakening (always between 07:00 h and 08:00 h, and approximately at 13:00 h (before lunch) and 20:00 h (before dinner). To maximize compliance with salivary collection times, all participants were asked to send both available coauthors (F.R.P., F.S.) a text message at each time point scheduled on the collection day. The day after home saliva collection, subjects returned the samples to the outpatient clinic, and they underwent a further SD-OCT examination to confirm the presence of CSC at the time of salivary collection.

After their initial enrollment in the study, two subjects (one control and one of the CSC group) were excluded because they incorrectly collected the salivary samples and because of occasional taking of drugs that were not allowed. Therefore 17 subjects per group were finally included in the study.

### Saliva Collection and Biomarker Assay

Saliva was collected using the Salivette sampling device (SciMart, St. Louis, MO, USA) and recovered by centrifugation at 3,000 rpm for 15 min. The unstimulated saliva flow rate (mL/min^−^^1^) was determined by dividing the volume of saliva by the collection time. Under basal conditions, the rate of saliva production was 0.5 mL/min^−1^. The saliva flow rate of valid samples should not be <0.1 mL/min^−1^.^42^ Samples were frozen at −20°C until the analysis was performed using commercially available assay kits for salivary α-AMY (Demeditec-Diagnostic, Kiel, Germany). The results are computed in units of α-AMY/mL saliva.

### Statistics

The statistical analyses and data visualization were performed by the SigmaPlot-11 software package (SxST.it, Italy). All quantitative variables were reported in the results as the mean and SE, unless otherwise specified.

For each subject, the diurnal salivary α-AMY production patterns were assessed by calculating the AUC_G_ using the trapezoidal method based on the three values measured during the experimental day (at 8.00, 12.00, and 20.00) and expressed as U/mL/h of α-AMY produced^26^^,^^43^; furthermore, raw biomarker data were used to estimate the variation between morning (at approximately 08:00 h, one hour after awakening, always before breakfast) and evening (at 20:00 h, always before dinner) in the production of α-AMY, applying the formula [(evening – morning)/morning] × 100.

To test for normality of distribution and homogeneity of variance, the Kolmogorov-Smirnov test was applied prior to statistical analyses. The two independent variable comparisons in the study population were performed with Student's *t*-test for continuous variables approaching a normal distribution and by the Mann-Whitney U test for continuous nonnormally distributed variables.

Pearson's correlation test was used to show any correlations between variables, and multiple linear regression analysis was used to predict dependent variables. The statistical significance was set at *P* < 0.05.^44^

## Results

### Demographic and Clinical Characteristics of the Study Population

The demographic and clinical characteristics across the study population groups at the time of enrollment are presented in Table 1. All subjects were Caucasian males. The baseline clinical data of patients were compared with those of the controls: no differences in age, educational level or sleep duration were found. The control and CSC groups did not show statistically significant differences in body mass index or basal cardiovascular parameters (systolic blood pressure; diastolic blood pressure; and heart rate), which were within the normal range in the study population.

**Table 1.** Demographic and Clinical Characteristics of the Study Population

	Control (n = 17)	CSC (n = 17)	Statistics
Age, years	50 (11)	48 (7)	NS
Weight, kg	79 (10)	83 (16)	NS
Height, cm	180 (8)	178 (7)	NS
BMI, kg/m^2^	29.4 (5.4)	27.5 (7.3)	NS
Educational level, years	14.8 (2.9)	13.3 (3.1)	NS
SBP, mm Hg	127 (9)	128 (5)	NS
DBP, mm Hg	79 (4)	82 (5)	NS
HR, beats/min	71 (6)	72 (4)	NS

BMI, body mass index; HR, heart rate; SBP, systolic blood pressure; DBP, diastolic blood pressure; NS, not statistically significant.

Data are expressed as the mean and (SD). Statistics: Two-group comparisons were performed with Student's *t*-test for continuous variables approximating a normal distribution or with the Mann-Whitney U test for continuous nonnormally distributed variables.

Finally, mean BCVA was 84.8 ± 2.1 letters in the CSC group and 87.1 ± 2 letters in the normal subjects. All the eyes were phakic with clear lenses. The subretinal fluid was present in all subjects with acute CSC. In 10 eyes the sub retinal fluid was included in the central subfield, while in the remaining eyes the sub retinal fluid was present in the inner ring.

### Choroidal and Choriocapillaris OCTA Features in the Study Population

**Figure 1.**
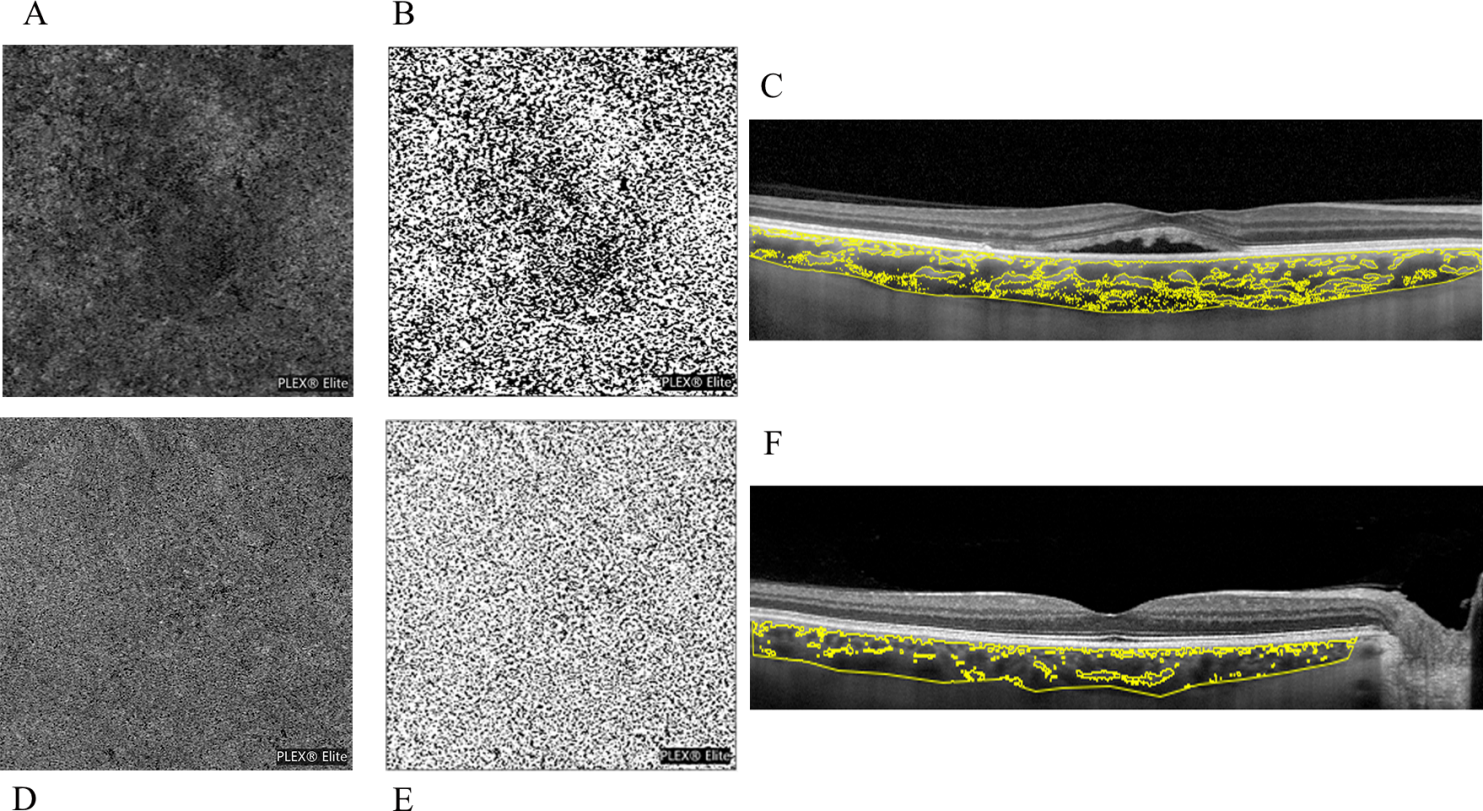
(**A**) OCTA scan of a CSC eye after choriocapillaris segmentation and exportation from the PLEX Elite 900 device, showing hyporeflective flow signal voids area. (**B**) The choriocapillaris images binarized with the Phansalkar method for the quantification of flow signal voids by using ImageJ (public domain software). (**C**) Enhanced depth optical coherence tomography images highlighting the CVI. Image binarization was performed by using Niblack autolocal threshold with ImageJ (public domain software) to calculate the total, luminal and stromal choroidal areas, respectively (TCA, LCA and SCA), the CVI was calculated as the ratio between LCA and TCA. (**D, E,** **F**) Images in the healthy eye, respectively.

The comparison of imaging biomarkers between the study eye and control eye is reported in Table 2. Eyes with CSC presented significantly higher FCT and CVI values than healthy control eyes. The evaluation of OCTA images of the choriocapillaris revealed that the prevalence of flow signal void area was higher in the CSC group than in the control group. Representative images of CSC and healthy eye, showing hyporeflective flow signal voids area and the choroidal vascularity index are reported in Figure 1.

**Table 2.** Comparison of Choroidal Vasculature Metrics and Choriocapillaris Flow Features Between CSC and Control Eyes in the Study Population

	Control Eye (n = 17)	CSC Study Eye (n = 17)
FCT, µm	254 ± 13.1 (53.9)	394 ± 23.3 (96.03)^*^
CVI	62.26 ± 0.5 (2.28)	76.45 ± 0.8 (3.45)^†^
Flow signal void area (%)	31.92 ± 1.88 (7.77)	36.96 ± 1.60 (6.60)^‡^

The data are expressed as the mean values ± SE [SD]. Two-group comparisons were performed with the Mann-Whitney U test for continuous nonnormally distributed variables.

* U = 29,000, T = 182,000, *P* < 0.001.

† U = 47,000, T = 200,000, *P* < 0.001.

‡ U = 92,000, T = 245,000, *P* = 0.073.

### Salivary α-AMY Diurnal Patterns in the Study Population

The diurnal α-AMY AUC_G_, which summarizes repeated diurnal salivary α-AMY measurements (08:00 h, 12:00 h, 20:00 h) in the study population, is reported in Table 3. The diurnal α-AMY AUC_G_ in the CSC group was significantly higher than that calculated in the control group. Furthermore, Table 3 reports the morning vs evening diurnal percentage variation of salivary α-AMY production calculated in the study subjects: Patients showed a significant reduction, close to 30%, of the expected increase in the evening production of α-AMY as detectable in the control group.

**Table 3.** Patterns of Salivary α-AMY Diurnal Production in the Study Population

Variable	Control (n = 17)	CSC (n = 17)
Salivary α-AMY diurnal AUC_G_ (U/min/mL)	1442 ± 99.9 [412]	1782 ± 107.2 [442]^*^
Salivary α-AMY diurnal percentage variation (%)	233 ± 31 [131]	66.7 ± 4.1 [17]^†^

The data are expressed as the mean values ± SE [SD].

* Comparisons performed with Student's *t*-test for continuous variables approximating a normal distribution (t = −2.319, *P* = 0.027)

† Comparisons were performed with the Mann-Whitney U test for continuous nonnormally distributed variables (U = 26,000, T = 416,000, *P* < 0.001).

### Correlations Between Salivary α-AMY Diurnal Production Patterns and Choroidal Parameters in the Study Population

**Figure 2.**
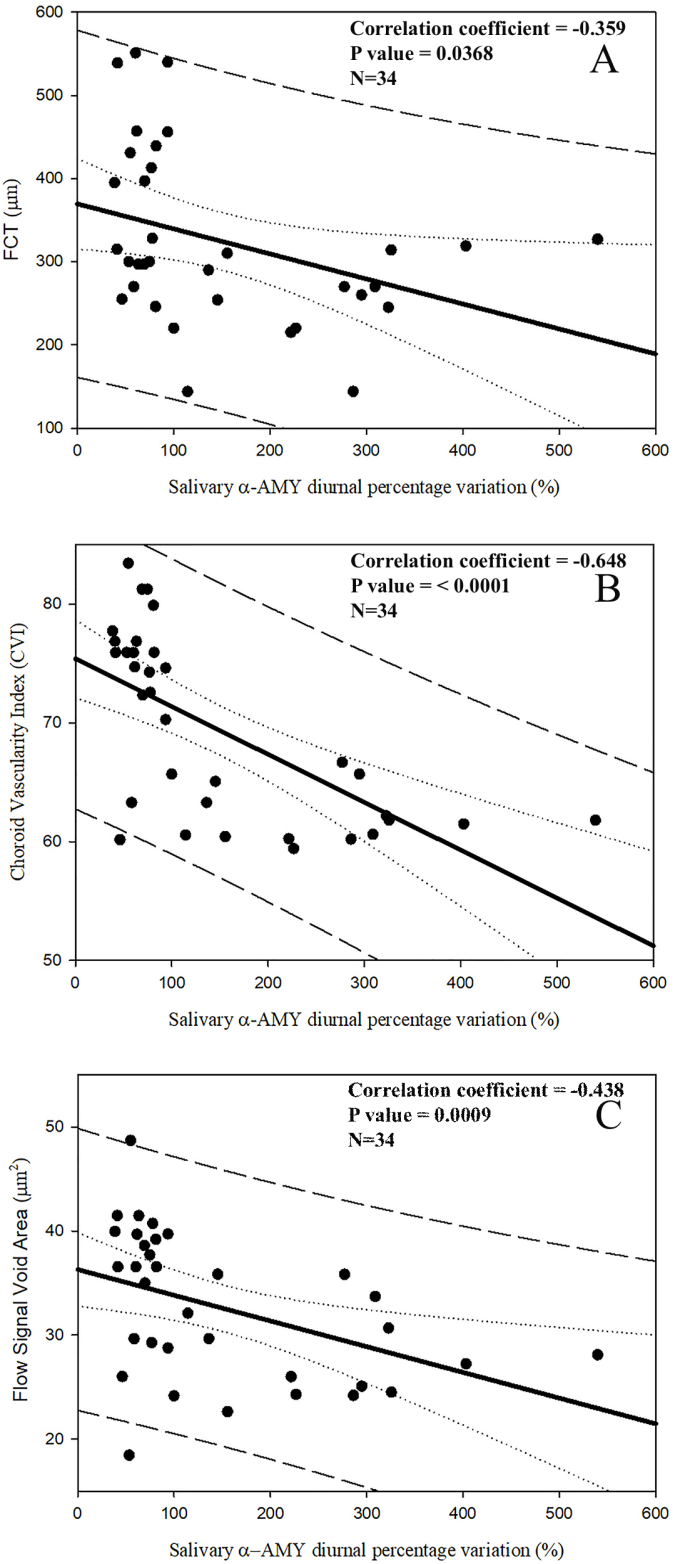
Scatterplots showing the relationship between salivary α-AMY diurnal percentage variation and the selected biomarkers of central serous chorioretinopathy imaging. *Continuous lines* represent best-fit linear regression; *dotted lines* represent confidence bands; *long dashed lines* represent prediction bands.

Figure 2 depicts scatterplots showing the statistically significant correlation between salivary α-AMY diurnal percentage variation and choroidal-retinal imaging features (FCT, CVI, flow signal void area). In contrast, no statistically significant correlation was found between salivary α-AMY AUC_G_ and choroidal-retinal characteristics in the study population (graphs not shown).

A multiple linear regression analysis was run to determine whether independent variables (salivary α-AMY AUC_G_ and salivary α-AMY diurnal percentage variation) were able to predict FCT, CVI and flow signal void area. As reported in Table 4, salivary α-AMY diurnal percentage variation was the sole statistically significant predictor of CVI and flow signal voids. Conversely, the AUC_G_ independent variable does not appear to account for the ability to predict any of the imaging features selected in the present study. Furthermore, none of the independent variables appear to account for the ability to predict FCT.

**Table 4.** Multiple Linear Regression Analysis Using the Independent Variables of Salivary α-AMY Diurnal % Variation and Salivary α-AMY Diurnal AUC_G_ to Predict the Dependent Variables of FCT, CVI, and Flow Signal Void Area in the Study Population

	β-coefficient	SE of β-coefficient	*t*	*P* Value
FCT^*^				
Salivary α-AMY Diurnal % Variation	−0.293	0.148	−1.985	0.056
Salivary α-AMY Diurnal AUC_G_	0.00628	0.0406	0.155	0.878
CVI^†^				
Salivary α-AMY Diurnal % Variation	−0.0376	0.00884	−**4.253**	**<0.001**
Salivary α-AMY Diurnal AUC_G_	0.00233	0.00242	0.961	0.344
Flow signal void area^‡^				
Salivary α-AMY Diurnal % Variation	−0.0235	0.00958	−**2.449**	**0.020**
Salivary α-AMY Diurnal AUC_G_	0.00107	0.00263	0.408	0.686

* R = 0.360, Rsqr = 0.130, Adj Rsqr = 0.0738.

† R = 0.661, Rsqr = 0.437, Adj Rsqr = 0.401.

‡ R = 0.443, Rsqr = 0.196, Adj Rsqr = 0.144.

## Discussion

In this study, adult male patients with naïve acute CSC underwent EDI-OCT scans followed by SS-OCTA, showing that they had increased FCT and CVI, as well as a higher distribution of flow signal void areas in comparison with matched controls, confirming and providing additional evidence that CSC may be caused by increased hydrostatic pressure in the choroid.^45^

Moreover, two main measures assessing diurnal salivary α-AMY secretion patterns were evaluated in the study population for their relationship with acute CSC-related choroid morphology parameters: raw biomarker data over time were used to estimate the AUC_G_ values with respect to baseline, which summarized three repeated salivary biomarker measurements from 08:00h to 20:00h. The diurnal α-AMY AUC_G_ values were higher in the CSC group than in the control group, signifying the overall increases in ANS activity was associated with the acute CSC.^14^

Furthermore, salivary α-AMY Diurnal percentage variation, computed as summary index of the circadian variation of the ANS activity, showed a CSC-related significant reduction of the increase in the evening production of α-AMY, which might reflect CSC-related dysregulation of ANS and actually detectable as expected in the control group.^13^^,^^14^

However, the key finding of the present study is that statistically significant correlations were selectively demonstrated between the salivary α-AMY diurnal percentage variation and the imaging features CSC-related (FCT, CVI and flow signal void area) measured in the study population. Furthermore, we have shown that salivary α-AMY diurnal percentage variation was the sole statistically significant predictor of CVI and flow signal void area in the study population. We are not aware of previous studies highlighting such a strict relationship between salivary α-AMY circadian secretion patterns and imaging features of the choroidal vasculature and choriocapillaris flow.

Under basal nonchallenging conditions, the protocol of the present study provided a multiple-time-point assessment of salivary α-AMY production characterizing the activity of the ANS system not directly related to the physiopathology of the stress response in acute CSC. In fact, organisms not only regulate their physiological functioning in response to acute challenges but also rely on biological rhythms to continuously adapt to the environment. Thus alterations in biological rhythms might impair health and well-being.^46^ From a pathophysiological point of view, the aforementioned anomalies affecting salivary α-AMY diurnal secretion patterns in our study population are consistent with the hypothesis of a dysregulation of ANS activity, indicative of an increased sympathetic drive affecting choroidal blood flow or RPE function and possibly even responsible for many of the psychophysical symptoms experienced by CSC patients.^9^^,^^47^^–^^49^

Recently with the present work, Scholtz and coworkers have hypothesized and demonstrated that salivary amylase can be a quantitative measurement of sympathetic activity in patients with active CSC,^50^ a concept considered rational and attractive in an interesting editorial commentary.^51^

Furthermore, it has been recently shown that exogenous adrenergic modulators may have a role in the pathophysiology of choriocapillaris function in CSC patients; however, the exact mechanism of action still needs to be ascertained.^52^^–^^54^ The role of sympathetic neurons in the choroid is likely to prevent overperfusion during times of increased sympathetic activation:^55^ during intense physical exercise, CSC patients appear to have a choroid particularly vulnerable to variation in systemic hemodynamic stressors showing a pathologically increased thickness, namely, an inability to regulate vascular tone, in response to increased perfusion pressures.^56^

The subjects enrolled in the present study had no signs of obesity (body mass index < 30) or hypertension thus avoiding the possible confounding effects of these comorbidities.^21^^,^^27^^,^^56^ Under basal nonchallenge conditions, the control group of the study population showed the expected significantly higher activity of α-AMY in the evening than in the morning.^25^ However, a potential limitation of the present study might be that the results cannot be directly generalized because our collection was from a population of males aged 40 to 60 years, despite having homogeneous demographic profiles. Future research will be necessary to confirm the present results in women, as well as elderly people, and extend the assessment to different forms of CSC, such as chronic or recurrent CSC.

In the present study, growing evidence has further underscored that measuring salivary α-AMY diurnal production patterns could be a specific and sensitive method for the determination of ANS dysregulation in individuals with CSC, because the circadian variation in ANS activity correlates with OCTA imaging metrics and is even predictive of CVI and flow signal void area.^20^^,^^21^^,^^49^^,^^57^^,^^58^ However, there could still be some questionable methodological aspects, because the sampling of saliva for biomarker measurement in this study occurred on only a single experimental day, preventing any day-to-day variability from being taken into account.^59^^–^^61^ Despite these limitations, our study had many strong methodological aspects, including that we explored the ANS activity in CSC under basal nonchallenge conditions by monitoring circadian changes in the salivary α-AMY secretion patterns across multiple time points, a more reliable approach than single-point sampling.^43^

In conclusion, the present study accurately described CSC-related effects on the ANS, adding novel information to the growing body of data suggesting that the dysregulation of the diurnal salivary α-AMY (diurnal percentage variation, ANS-associated) is present in CSC in susceptible individuals, allowing us to observe a causal link between ANS derangement and choroidal acute CSC features. As a whole, we believe that the dysregulation of the functional chronobiological adaptive mechanism causing the failure of essentially ANS-related, not limited to a single time point measurement but actually scheduled at several times points, should be included among the trigger factors of some of the acute CSC features,^13^^,^^50^^,^^51^ providing ophthalmologists with a physiopathological therapeutic target to be explored also in the field of preventive medicine.
